# Rational Discovery of Antimicrobial Peptides by Means of Artificial Intelligence

**DOI:** 10.3390/membranes12070708

**Published:** 2022-07-14

**Authors:** Paola Ruiz Puentes, Maria C. Henao, Javier Cifuentes, Carolina Muñoz-Camargo, Luis H. Reyes, Juan C. Cruz, Pablo Arbeláez

**Affiliations:** 1 Center for Research and Formation in Artificial Intelligence, Universidad de los Andes, Bogota 111711, Colombia; p.ruiz@uniandes.edu.co; 2 Department of Biomedical Engineering, Universidad de los Andes, Bogota 111711, Colombia; jf.cifuentes10@uniandes.edu.co (J.C.); c.munoz2016@uniandes.edu.co (C.M.-C.); 3 Grupo de Diseño de Productos y Procesos (GDPP), Department of Chemical and Food Engineering, Universidad de los Andes, Bogota 111711, Colombia; mc.henao10@uniandes.edu.co (M.C.H.); lh.reyes@uniandes.edu.co (L.H.R.)

**Keywords:** antimicrobial, peptides, artificial intelligence, graphs, molecular dynamics

## Abstract

Antibiotic resistance is a worldwide public health problem due to the costs and mortality rates it generates. However, the large pharmaceutical industries have stopped searching for new antibiotics because of their low profitability, given the rapid replacement rates imposed by the increasingly observed resistance acquired by microorganisms. Alternatively, antimicrobial peptides (AMPs) have emerged as potent molecules with a much lower rate of resistance generation. The discovery of these peptides is carried out through extensive in vitro screenings of either rational or non-rational libraries. These processes are tedious and expensive and generate only a few AMP candidates, most of which fail to show the required activity and physicochemical properties for practical applications. This work proposes implementing an artificial intelligence algorithm to reduce the required experimentation and increase the efficiency of high-activity AMP discovery. Our deep learning (DL) model, called AMPs-Net, outperforms the state-of-the-art method by 8.8% in average precision. Furthermore, it is highly accurate to predict the antibacterial and antiviral capacity of a large number of AMPs. Our search led to identifying two unreported antimicrobial motifs and two novel antimicrobial peptides related to them. Moreover, by coupling DL with molecular dynamics (MD) simulations, we were able to find a multifunctional peptide with promising therapeutic effects. Our work validates our previously proposed pipeline for a more efficient rational discovery of novel AMPs.

## 1. Introduction

Antibiotics have revolutionized medicine since their discovery in 1911, decreasing morbidity and mortality rates of multiple diseases [1]. However, the emergence of resistant microorganisms has led to antimicrobial resistance (AMR), becoming a public health problem of increasing concern in recent years. AMR negatively impacts population health, healthcare systems costs, and gross domestic product (GDP) worldwide [2]. AMR is thought to be the result of misuse and overuse of antibiotics, self-medication, self-interrupted treatments, genetic plasticity, and sheer dogged adaptability of the microorganisms themselves [3,4]. For the United States, the CDC reported that in 2019, 2.8 million resistant bacterial infections resulted in 35,000 deaths. The cost associated with these incidents was approximately USD $20 billion, representing a growth of 570% of reported cases compared to 2016 [5,6]. Due to the rapid generation of AMR to newly discovered or designed antimicrobials, the pharmaceutical industry has virtually stopped its search and investment in this field [7]. By 2015, 15 out of 18 of the largest pharmaceutical companies had abandoned the search for novel antibiotics [7]. Recent studies have estimated that, without new and more potent AM molecules, by 2050, resistant pathogens will claim more than ten million deaths per year, with a higher prevalence in developing countries and an associated cost of about USD $66 trillion [8].

Antimicrobial peptides (AMPs) have been studied during the last decade as an alternative treatment, as they have been reported to overcome the resistance mechanisms of an ample variety of microorganisms [9]. AMPs represent essential components of the higher organism’s innate immunity with a broad spectrum of antimicrobial activities. Additionally, they exhibit diverse chemical characteristics and varied target cells, acting through multiple mechanisms depending on their topology, propensity for aggregation, and lipid interactions with cellular membranes [10]. Those characteristics make them promising agents with lower chances of acquiring resistance [9,11]. Furthermore, a common feature in AMPs, as for other peptides and proteins, is their multifunctionality, which indicates that AMPs can generate multiple physiological outcomes by targeting different intracellular components [12,13]. Polypharmacological peptides have become an area of increased interest for researchers in recent years because of their multiple applications in tissue engineering [14,15], and their proven effectiveness as treatments for numerous diseases [13,16,17,18,19,20].

Several functions in a single antimicrobial peptide have high medical potential as they might be helpful to increase specificity, facilitate organelle targeting, or enable cargo delivery. Specifically for AMPs, high binding affinity towards DNA and the capability of penetrating cellular membranes (CPPs) have been reported as critical features to enable superior therapeutic performance [12]. Among many others, peptides with antimicrobial and cell-penetrating capabilities might serve as enablers for the treatment of different types of cancer, obesity, neurodegenerative diseases and even serve as key agents for gene-delivery therapies [12,21]. Therefore, AMPs and multifunctional peptides (AMPs & CPPs) are of great interest to the pharmaceutical industry and its allies, given that they offer a much ampler number of routes for the recovery of their research investment. However, the discovery of such molecules is a tedious, costly, and time-consuming task since it involves the in vitro screening of large libraries of randomly or rationally designed sequences [22,23].

Chemoinformatic models were designed to reduce the time and costs associated with discovering novel AMPs. Nevertheless, their accuracy has been insufficient to improve the process [24]. As an alternative, deep learning (DL) techniques have emerged with promising preliminary results for sequences’ analysis through molecular representations built aided by neural networks (NNs) [25]. The main differences between chemoinformatic tools and DL approaches are related to how each model represents the molecules. While the former uses manually designed representations, such as different fingerprints or physicochemical descriptors designed by experts [24], the latter learns optimal representations by jointly extracting and analyzing several features that an expert may disregard while performing the task manually [26].

Traditional chemoinformatic approaches are more problematic to apply for multiple molecules due to the hand-crafted features and because exploring large databases becomes a highly tedious and time-consuming task. In contrast, once trained, DL models can analyze millions of molecules simultaneously to identify the most promising candidates in only a few minutes with relatively short post-processing times. Therefore, DL models are thought to reduce the time and costs associated with discovering AMPs by identifying the most promising candidates more accurately, which likely leads to a significant reduction in the number of experiments [27].

Peptides’ discovery and analysis through artificial intelligence started in the 1990s; nonetheless, literature reports only a few studies for antimicrobials based on deep learning algorithms. These works have mainly implemented recurrent neural networks (RNNs) initially designed for natural language processing [28]. Peptide sequences share common features with a natural language, such as the essential information related to the order of the characters and their corresponding correlations. Therefore, RNN might be well-suited for analyzing atom sequences, which is fundamental to understanding the potential bioactivity of each peptide sequence [29]. The design of neural network-based algorithms for peptides involves developments on two fundamental fronts: (i) the representation of the peptides and (ii) the architecture used for each representation.

Vectors are commonly used as representations to preserve the order of amino acids. In some research reports, peptides have been represented as 20 × M one-hot vectors where each row is intended to describe the presence of one of the 20 essential amino acids in the sequence. At the same time, M is the entire length of the molecule [30,31]. Alternatively, 1D 1 × M vectors have been considered, where each amino acid in the sequence is encoded by a number from 0 to 19 [32]. More recently, peptides have been reproduced with the aid of the word2vect embedding [33]. However, these representations disregard the interactions between amino acids and the atoms within each amino acid. In addition to the sequential representation, essential properties of AMPs must be computed to make the vectors more descriptive. These include amino acid composition, composition-transition-distribution, overall PseAAC, and the reduced amino acid composition pseudo-K-tuple [34].

Regarding the architectures, both long-short term memory (LSTMs) and gated recurrent units (GRUs) cells have been used for AMPs discovery. LSTM cells have been used together with convolutional layers as feature extractors [32] or with attention and context layers to extrapolate concepts learned in transformers [30]. In contrast, GRU cells have been used with word2vect to generate trigrams of consecutive amino acids, successfully discovering non-homologous peptides [33]. However, DL models are usually trained on small databases, which implies a lower generalization power when it comes to assigning features to the new data. Additionally, each research task usually designs its database to develop the algorithms, and evaluation metrics vary significantly within the field. In this regard, source codes are rarely publicly available; therefore, comparison within methods to evaluate the advance of new algorithms is currently a significant challenge.

**Figure 1:**
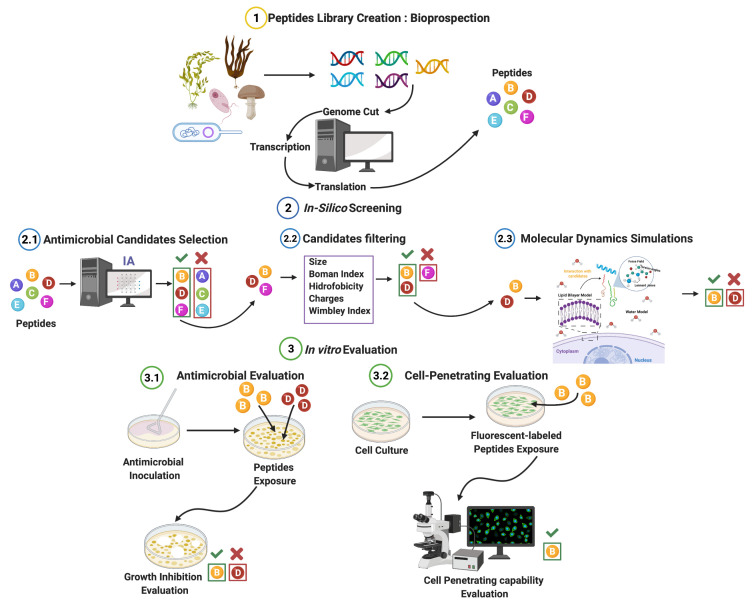
AMPs rational discovery pipeline. (1) A peptide library was generated by cutting the *Escherichia coli* genome in silico. (2.1) The improved DL algorithm analyzes the library to select promising candidates with membrane activity. (2.2) Candidates were filtered using physicochemical properties to obtain viable AMPs. (2.3) Molecular Dynamics was implemented to find candidates exhibiting cell-penetrating capability. (3.1) AMPs candidates were evaluated in vitro to obtain the MIC. (3.2) Peptides with additional cell-penetrating activity were evaluated in mammalian cells via confocal microscopy. Created with biorender.com.

The work presented here follows our proposed pipeline for rational discovery [35], focusing on the DL model. Briefly, we implemented DL techniques to find new AMPs sequences with potentially high antimicrobial activity, followed by analyzing the candidates’ interaction with a model cell membrane via molecular dynamics (MD) to select those that also show potent cell-penetrating capabilities and, therefore, attractive multifunctionality. Furthermore, different from most currently available models, our algorithm is not only capable of classifying antimicrobial from non-antimicrobial but also of classifying them into the four main microorganisms’ including, antibacterial, antiviral, antifungal, and antiparasitic. Subsequently, we validated the antimicrobial and cell-penetrating activity in vitro. Our whole pipeline is illustrated in Figure 1. To overcome the limitations of the existing DL methods, we proposed evaluating graph representations, which have been primarily disregarded for this type of study and enable us to put into consideration the interaction (i.e., bonds) between the amino acids and not only their primary sequence (1D representation), as has been the case of most previous works. Furthermore, due to the non-Euclidean nature of peptides, graph representations enable accurate and explicit modeling of atoms’ and bonds’ spatial configurations, enabling learning from the most basic information of the peptide molecules, as proven by [36] for organic molecules.

Additionally, we extensively collected 19 publicly available databases to ensure a much more robust feature generalization to the newly incorporated data, which resulted in around 25,000 peptides to train the DL models. To the best of our knowledge, our work encompasses the most extensive AMPs recollection reported thus far. Finally, the proposed pipeline was evaluated with a new peptide library generated by cutting in silico the genome of *Escherichia coli* with restriction enzymes and subsequently translating the obtained fragments with the aid of all the possible reading frames.

## 2. Materials and Methods

### 2.1. Database

As mentioned above, one of the main limitations of the existing AI methods for AMPs prediction is the quantity and diversity of data used for training the models, which implies lower generalization power of crucial properties to newly added data [26]. Therefore, an exhaustive search was performed over 19 public available databases (Table 1).

Before processing the sequences, 44,762 peptides were collected. However, filtering was performed to ensure that all data would be useful for the IA methods’ training. The processing is detailed below:

1. Peptides with synthetic modifications were deleted.
2. Peptides with unknown amino acids (X) within their sequence were deleted.
3. Peptides with pyrrolysine (O), Selenocysteine (U), β-Alanine (Bal), 3-Naphthylalanine (Nal), and 2-Aminobutanoic acid (Abu) were deleted.
4. Peptides with J (leucine or isoleucine) were maintained, considering both amino acids.
5. Peptides with B (aspartic acid or Asparagine) were maintained, considering both amino acids.
6. Peptides with Z (Glutamic acid or Glutamine) were maintained, considering both amino acids.
7. Duplicated sequences were deleted while preserving all their associated activities.

The database consists of 23,967 unique sequences with 38,924 labels associated with 28 biological activities. Table 2 shows their relative abundance within the database.

**Figure 2:**
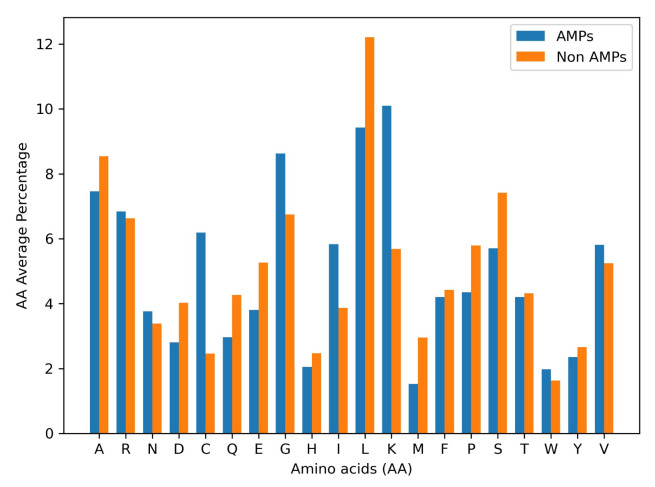
Amino acids distribution. Abundance of each amino acid within all sequences of AMPs and Non-AMPs.

Antimicrobial peptides represent 55.78% of all data and, in turn, 4303 are antibacterials, 4006 antivirals, 2233 antifungals, and 248 antiparasitics. The remaining sequences have no reported specific activity. Their average size is 28 AA, with lengths varying from 2 to 344 AA. Likewise, the average size of non-AMPs is 24 AA. Figure 2 shows the amino acid composition of AMPs vs. Non-AMPs. As expected, AMPs are richer in lysine (K) and cysteine (C) compared with non-AMPs. On the contrary, arginine (R) and tryptophan (W), which are related to AM activity by enabling electrostatic interactions and hydrogen-bonding potential, have similar abundances in both types of peptides [55]. The homogeneous distribution of AAs and sizes for both types of peptides are well-suited for DL techniques, as they will need to learn underlying physicochemical cues to predict the peptides’ biological activity correctly.

This research proposes both a rough and a fine-grained problem. The former relates to the prediction of antimicrobial and non-antimicrobial peptides. In contrast, the latter relates to the classification of peptides within the four principal classes of AMPs: antibacterial, antiviral, antifungal, and antiparasitic.

#### 2.1.1. AM Prediction

Dataset partition involved labeling all peptides with no antimicrobial activity as non-antimicrobial, which led to 13,468 AMPs and 10,499 non-AMPs. Both types of peptides were divided into 80% train and 20% test, considering both length and amino acid distribution within the sequences. Furthermore, the train set was split into four folds for cross-validation purposes. AP is reported over the ensemble of the four models.

#### 2.1.2. Fine AM Prediction

The four main classes of AMPs are Antibacterial (AB), Antiviral (AV), Antifungical (AF), and Antiparasitic (APT). We have 4303 AB, 4006 AV, 2233 AF, and 248 APT within the dataset with 7393 unique sequences. All peptides were divided into 80% train and 20% test, considering length, AA distribution, and antimicrobial activities. Moreover, the train set was split into four folds to perform cross-validation. The normalized average precision (NAP) is reported over the ensemble of the four models.

### 2.2. AMPs-Net

**Figure 3:**
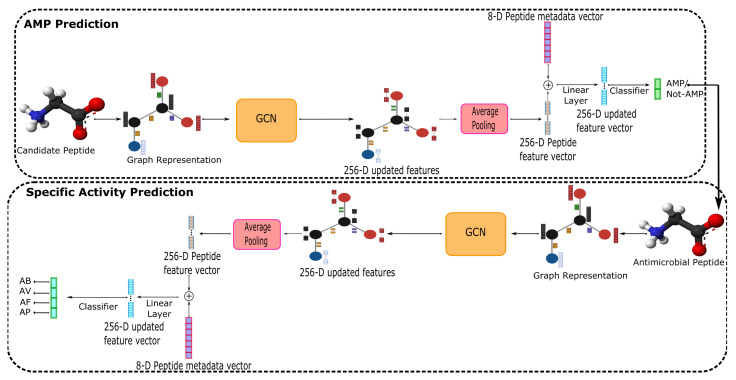
AMPs-Net overview. The FASTA sequence of a candidate peptide was transformed into a graph representation and used as input to a Graph Convolutional Neural Network. Based on the message-passing algorithm, a 256-Dimensional updated graph was obtained and averaged over the feature dimensions. The representative vector was concatenated with the physicochemical properties of the peptide, and a linear layer was then used to classify the peptide into an AMP or Non-AMP. Peptides predicted as AMPs were further analyzed by a similar network that outputs the probability of finding them within the four sub-classes of antimicrobial peptides.

AMPs-Net builds upon previous work [36], a deep message-passing framework optimized to predict target–ligand interactions. As shown in Figure 3, AMPs-Net comprises two sequential modules. The first classifies the peptides between AMP and Non-AMP, while the second predicts the AMPs probability towards the four main types of antimicrobial activities.

The main component of both AMPs-Net modules is a Graph Convolutional Network (GCN), which enables the analysis of non-euclidean data, such as that of chemical compounds that need to preserve the spatial configuration in a 2D space. Peptides’ graph representations were constructed from the FASTA sequences. Given a peptide, its graph was represented as $G=(V,E,X_{v},X_{e})$, where V denotes the set of nodes (atoms), E the set of edges (bonds), $X_{v}$ the set of atom features and $X_{e}$ the set of bond features. The atom feature vector $x\in X_{v}$ contains nine properties of the atom v∈V, which are shown in Table 3.

**Figure 4:**
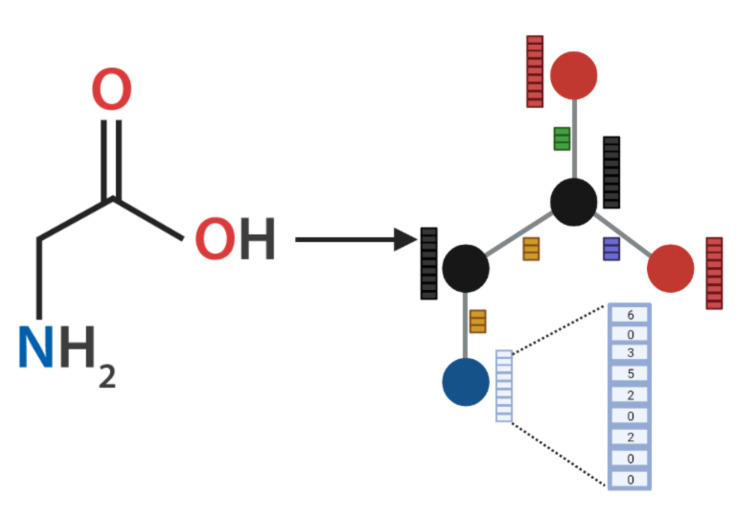
Graph Representation of Glycine. In the graph representation of molecules, atoms are represented as nodes and bonds as the edges. Each amino acid atom is represented within the graph by nine physicochemical properties (Table 3). Likewise, bonds between the atoms were described by three properties. The same color implies the same atom and/or bond and, therefore same feature vector. Created with Biorender.com.

The bond feature vector $x_{vu}\in X_{e}$ was built from three characteristics of the bond $e_{vu}\in E$ between atom *v* and atom *u*, also shown in Table 3. Moreover, all bonds were assumed bidirectional, $X_{e_{vu}}=X_{e_{uv}}$. Glycine graph representation is shown in Figure 4, where it can be seen that such graph representation for molecules usually disregard the hydrogen atoms within the molecule.

The GCN modules comprises a message-passing framework designed for molecular property prediction [56]. Being $N_{v}$ the set of neighbors of atom *v*, the message passing algorithm is described by message construction (Equation (1)), message aggregation (Equation (2)) and node update (Equation (3)) equations.

(1) $$M_{vu}=\rho \left(X_{v},X_{u},Xe_{vu}\right),u\in N_{v}$$

(2) $$M_{v}=\zeta \left(\left\{M_{vu}|u,\in Nv\right\}\right)$$

(3) $${Xv}^{n}=\phi \left({Xv}^{n-1},M_{v}\right)$$

The message construction function ρ is applied over an atom’s, neighbor’s, and corresponding edge’s features to obtain an individual message for each neighbor node. The message aggregation function (ζ) takes all the individual messages of the neighbor nodes and outputs one aggregated message. Finally, the node update function (ϕ) updates the node’s features using the aggregated message. Once all the update layers were completed, the updated graph (i.e., atom) contains information about the local regions of the molecule.

Overall, AMPs-Net generates a graph representation of each peptide from its corresponding FASTA sequence. Peptides graphs were then used as input for the GCN module with 20 message-passing layers, a softmax as aggregation function, and 4-layers MLP as update function. The GCN module follows an unconventional order, first the batch normalization layer, followed by a ReLU activation, then a dilated message passing layer, and finally, the addition of residual connections. The final updated graph has feature vectors of 256 in size for each atom and bond. An average pooling over the atom’s features was performed to obtain a unique representation for each peptide. This representation was concatenated to a metadata vector with eight peptide physicochemical properties and was used as input to a linear layer that outputs a new 256-D vector. This vector was subsequently employed for binary or multiclass classification, AMP prediction for the coarse task, and the probabilities for each AMP activity for the fine task. AMPs-Net was trained in 1 GPU QuadroRTX8000 of 48 GB for 160 epochs with a batch size of 112; a learning rate of $5\times 10^{-5}$, and an adamax optimizer.

### 2.3. AMP Candidates

Escherichia Coli (*E. coli*) genome was cut in silico by the restriction enzyme Sau3IA, which can be replicated experimentally to build a peptide library in vitro for screening purposes in future work. Furthermore, the restriction site of Sau3IA is often found within the genome, enabling a large number of peptide candidates [57]. All segments were transcribed and translated, assuming the presence of an initiating codon in all cases. Translation involves the use of multiple reading frameworks. Figure 1 (1.1) illustrates the entire process.

Once the probability scores for AMPs were obtained, the sequences were further filtered to obtain the most promising candidates. This was achieved by considering several properties to classify membrane-active peptides, including size, Boman Index, net charge at pH 7.4, hydrophobic ratio, hydrophobic moment, aliphatic index, instability index, and isoelectric point. Furthermore, as a negative control, we included AHB-1 (MFVFLVLLPLVS), a potent membrane-translocating peptide that exhibited no antimicrobial activity and was recently discovered by us from a comprehensive analysis of the spike protein of SARS-CoV-2 [58].

### 2.4. Molecular Dynamics Analysis

Some of the filtered peptide candidates were selected by size, net charge at pH 7.4, and Boman index to evaluate their cell-penetrating capabilities. The prediction of these peptides’ secondary and tertiary structures was carried out to assess the molecule’s biological activity after folding. This prediction was conducted in the I-Tasser server [59]. The server generates a top 5 predicted de novo structures in PDB format. The sequences with the highest C-score, representing the most accurate prediction, were selected for further studies.

MD simulations were carried out using the GROMACS version 2019.3 software with the semi-atomistic Force Field GROMOS96 53a6, modified to its correct usage with lipid membranes by adding the Berger lipid parameters [60]. A leap-frog integrator was used in all simulations, with an integration time step δt of 0.001 ps. Van der Waals and short-range electrostatic interactions cutoff were set at 1.2 nm, while long-range electrostatics were calculated by the Particle Mesh Ewald (PME) method. Finally, 3-D periodic boundary conditions were imposed.

#### 2.4.1. Non-Equilibrium Pulling (Flat-Bottom)

The simulation box (6.41840 × 6.44350 × 12.00000 nm) was built with a simplified eukaryotic cell membrane model (which was composed of 128 lipids of dipalmitoylphosphatidylcholine (DPPC)), water as a solvent, and the peptide to be evaluated located parallel to the membrane at a distance of 5 nm from the bilayer’s headgroups. Ions of Na${}^{+}$ or Cl${}^{-}$ were employed to assure the system’s electroneutrality. Subsequently, an equilibration of 50,000 steps was carried out at a constant temperature (323 K), using the modified Berendsen thermostat (V-rescale), and at a constant pressure (1 bar) by 50,000 steps according to the Parrinello-Rahman barostat, ensuring equal conditions for each component of the system. Lastly, energy minimization was done to obtain relaxed low-energy conformations to prevent significant steric hindrance limitations [61]. Once the system was correctly parameterized, position constraints were removed. The system was allowed to interact through a steered MD simulation where a flat-bottom potential of 2000 kJ/mol-nm${}^{2}$ was applied at 3.5 nm from the center of mass of the membrane. The simulation was run for 500 ns, which encompassed 250,000,000 steps.

#### 2.4.2. Non-Equilibrium Pulling (Umbrella SAMPLING)

Peptides with the most marked tendency to penetrate the lipid bilayer were chosen to determine their preferential location within the membrane by an Umbrella Sampling simulation. Each peptide was located at a distance of 6 nm from the bilayer’s headgroups in a simulation box of 13 nm in length. The system was solvated with water, and counterions were added for electroneutrality. Next, an NVT equilibration of 50,000 steps was conducted, followed by an NPT equilibration of 50,000 steps.

The free energy of the peptides through the lipid membrane was obtained from the Potential Mean Force (PMF) curve generated by the Umbrella Sampling simulation. This was accomplished by running a 65,000-step steered MD simulation right after equilibration. In this approach, the peptide was transferred from the bulk of the aqueous phase into the membrane under a harmonic potential of 600 kJ/mol-nm${}^{2}$. The simulations resulted in several configurations with an average distance of 0.2 nm between them. Finally, each configuration was taken as an independent simulation, balanced, and minimized again, and a production run of 5,000,000 steps was taken further. The PMF profile was obtained by applying a Weighted Histogram Analysis Method (WHAM).

#### 2.4.3. Behavior Inside Membrane

An orthorhombic simulation box was built with the membrane model at its center, and the peptide molecule was located vertically to the bilayer’s center of mass (COM). The system was parameterized as described previously, and finally, position constraints were removed, allowing the system to interact for 100 ns. The trajectories obtained enabled the extraction of structural analysis such as Root Mean Square Displacement (RMSD), Radius of Gyration, Average Mass Densities, and Interaction Energies.

### 2.5. In Vitro Validation

#### 2.5.1. Antimicrobial Activity Validation

Peptides’ antibacterial activity was evaluated for concentrations ranging from 250 μM to 0.12 μM in serial dilutions. The assay was performed as described previously by Perez et al. [62]. Briefly, Gram-positive *Staphylococcus aureus (S. aureus)* and Gram-negative *E. coli* were cultured at 37 °C in LB agar plates overnight, followed by a culture in fresh LB medium until they achieved a 0.5 value according to the McFarland standard. Cells were centrifuged at 3600 rpm for 5 min, washed three times with 2 mL of 10 mM Na${}_{2}$HPO${}_{4}$ buffer (pH 7.4) or NaCl 0.9% *w*/*v*, and diluted in the same solution to obtain a concentration of $10^{4}$ CFU/mL. CFU was calculated with the aid of the growth curve for each bacterial strain and its absorbance at 595 nm (Equations (4) and (5)).

(4) $$S.\;aureus\;UFC=\left(11.9*Culture_{Abs_{595}}-0.547\right)*10^{8}$$

(5) $$E.\;coli\;UFC=\left(7.97*Culture_{Abs_{595}}-0.367\right)*10^{8}$$

100 µL of samples were prepared at 1:1 ratio peptides:bacteria by triplicate in a 96-well microplate and subsequently incubated for 2 h at 37 °C. Next, 100 μL of LB medium was added to each well, followed by incubation at 37 °C for 18 h. The absorbance was measured at 595 nm to evaluate possible inhibitory growth effects. The negative control for this assay was buffer with LB medium and bacteria (without antibacterial activity), and the positive control was only buffer and medium (maximal antibacterial activity). The collected data was used to estimate the minimum inhibitory concentration (MIC).

#### 2.5.2. Synthesis of Low Molecular Weight Chitosan Nanoparticles (CNPs)

CNPs were synthesized following the ionic gelation method [63]. Briefly, 2.4 mg/mL of LMW Chitosan (50–190 kDa, deacetylation degree of 75–85%, CAS 9012-76-4) was dissolved in acetic acid 2% *v*/*v* under magnetic stirring for 3 h. This procedure protonates the amine groups of monomers and therefore increases its solubility. Afterward, to induce a partial charge restoration, the pH of the mixture was adjusted to 3.6. To obtain the CNPs, chitosan chains were crosslinked with 1.2 μL of glutaraldehyde per milliliter of chitosan, added dropwise, and left under stirring for 1 h. To purify the CNPs, the reaction mixture was dialyzed against Type II water at room temperature for three days using a 2 kDa membrane (Sigma-Aldrich, St. Louis, MO, USA). Lastly, the CNPs were lyophilized and stored at 4 °C.

#### 2.5.3. Functionalization of CNPs

CNPs (100 mg) were resuspended in 70 mL of type II water, mixed with 2 mL of glutaraldehyde 2% *v*/*v*, activating the CNPs surface for 1 h. Afterward, 1 mg of the peptide was added and left to conjugate under agitation for two days. Rhodamine B was used as a fluorophore to label the CNPs. Before conjugating to the CNPs, 7 mg of EDC and 5 mg of NHS were mixed in 5 mL of type II water until complete dissolution. Next, 200 μL of DMF and 6 mg of Rhodamine B were added to the mixture and left to react at 40 °C for 15 min. Afterward, this activated Rhodamine B was mixed with the CNPs-peptide nanobioconjugates and left under agitation for 24 h at room temperature. To remove unconjugated rhodamine B, the mixture was dialyzed against Type II water using a 2 kDa membrane (Sigma-Aldrich, St. Louis, MO, USA). Finally, the labeled nanobioconjugates were lyophilized and stored at 4 °C until further use.

#### 2.5.4. Cell Penetrating Activity Validation

The ability of immobilized peptides (CNPs-peptide nanobioconjugates) to translocate cell membranes and distribute intracellularly was assessed by estimating the surface area coverage after internalization into NHA cells (Lonza CC-2565). Endosomal escape after 3 h of exposure was estimated by calculating the colocalization between the nanobioconjugates and Lysotracker Green DND-26 (Thermo Fisher, Waltham, MA, USA) through the Pearson correlation coefficient (PCC). Imaging was conducted in an Olympus FV1000 confocal laser scanning microscope (CLSM) (40X/0.6 UCPlan FL N and PlanApo 60x/1.2 oil immersion objective, Olympus, FV1000). Briefly, 18,000 cells per well were seeded on a glass slide of 3.5 mm diameter previously coated with Poly-D-Lysine (Thermo Fisher (Gibco), Waltham, MA, USA). NHA cells were incubated in an ABM medium supplemented with 3% (*v*/*v*) FBS for 24 h (37 °C, 5% CO${}_{2}$) to allow cell adhesion. After incubation, cells were exposed to labeled CNPs-peptide nanobioconjugates for 3 h in a non-supplemented medium at 12.5 μg/mL concentration. Then, cells were exposed to an ABM solution with Hoechst 33342 (Thermo Fisher, Waltham, MA, USA) (1:10,000) and Lysotracker Green DND-26 (1:10,000) for 5 min before observation via confocal microscopy. Excitation/Emission wavelengths were set at 405 nm/461 nm, 488 nm/535 nm, and 559 nm/600 nm to detect nuclei, endosomes, and nanobioconjugates, respectively. Nanobioconjugates were compared with bare CNPs to evaluate the impact of the novel peptides on membrane translocation. Image analysis was performed with the Fiji-ImageJ software. Data analysis was completed using the GraphPad Prism V 6.01 software (GraphPad Software, La Jolla, CA, USA). Statistical comparisons were made using the unpaired *t*-test, and *p* ≤ 0.05 (*) results were considered significant. Data are given as average ± one standard deviation.

## 3. Results and Discussion

### 3.1. AMP Prediction

Table 4 compares binary AMPs-Net with four state-of-the-art deep learning methods. Pre-trained models on their original dataset or prediction servers were used to evaluate the performance of all the other methods in our test set. Even though this approach might have some advantages, we failed to obtain retraining methods in our dataset due to the lack of publicly available or non-working codes. Nevertheless, all the evaluated methods claim that their performance and generalization are high enough to be used as virtual-screening tools for peptides. Our method outperforms all of them by a margin that ranges from 8.8% to 19.02% in average precision (AP) and from 5.74% to 24.23% for accuracy (ACC). All methods considered for comparison are based on recurrent neural networks of both GRU and LSTM cells; however, the input representation of the peptides was different from ours. The boost in performance entails that our data recollection process and formulation of the problem through neural graph networks effectively enhanced the prediction capability for antimicrobial activity. Furthermore, it is essential to note that even though the AP is a more robust metric, we also calculated ACC given that both CAMP${}_{R3}$ and AMPDiscovery prediction servers failed to return the prediction probabilities.

AMPEPpy, one of the most recent Random Forest algorithms, was also evaluated in the AMPs prediction task. AMPEPpy consists of 128 decision trees with 105 encoded features for each peptide. Publicly available code-enabled training with our data. As shown in Table 4, both methods (AMPs-Net and AMPEPpy) have comparable performance, with AMPEPpy outperforming ours by a slight margin. Nevertheless, it is important to highlight that its developers stated that AMPEPpy’s performance is highly dependent on the training data, which implies that the prediction could be significantly compromised if the predicted peptides have a different distribution in length, AA composition, or physicochemical properties than the ones used for training purposes [68]. Furthermore, inference time with AMPEPpy is much slower than AMPs-Net’s, i.e., 326 s vs. 45 s. This indicates that the processing time increases about seven times compared to AMPs-Net. Conversely, our algorithm is likely much more suitable for extensive genome searches.

Table 5 shows the optimization process required for our binary AMPs-Net architecture. A deeper network was beneficial to learning peptide structures. This was expected as peptides exhibit larger structures compared to the small organic compounds that Deeper GCN was initially designed to process. Our data suggest that increasing the GCN layers from 10 to 20 enhances the performance; however, beyond such depth, the network’s learning ability is significantly impaired. This decrease can be explained by the considerably large databases needed to assure that deeper networks learn generalizable features instead of learning just their training dataset. In both cases (i.e., 25 and 30 layers), the AP over the training set is higher compared with the test set, demonstrating an over-fitting for the training set.

Increasing the hidden size (HS) of features for up to 256 consistently led to increased the network’s performance; however, a further increment failed to improve the AP significantly. However, under this scenario, the computational time required almost doubled. The number of multilayer perceptrons (MLPs) showed similar behavior with an increase in performance for up to 4MLPs. Finally, the essential physicochemical properties used to select the most promising AMPs were added to the model as metadata for each peptide. This extra data enhanced the performance even further.

The Antimicrobial Peptide Prediction module achieves a performance of 95.76% in Average-Precision (AP). This very high performance enabled us to use the model to perform virtual screening of the peptide library generated as described in Section 2.3. Furthermore, after achieving the best architecture for the binary task, we trained the same architecture for specific activity prediction; however, no increase in performance was obtained after further optimization. To the best of our knowledge, this DL method is the first to classify within the four main AMP classes with only one model. AMPs-Net specific activity prediction achieved 71.36% for the normalized AP (Table 6). Even though the performance is much lower than in the binary setup, the AP for antibacterial and antiviral classes were 90.67% and 84.54%, respectively. This performance was considered sufficiently high for the needs of the proposed virtual screening. On the contrary, antifungal and antiparasitic activities showed much lower performances, reaching 50.93% and 24.73%. The performance distribution correlated well with the number of samples per class in our database. Therefore, more antiparasitic and antifungal peptides validated experimentally will be needed in future work to increase the performance further.

### 3.2. Candidates Selection

Digestion and translation of *E. coli*’s genome with Sau3AI led to 423,697 sequences to analyze. Out of them, 2284 were predicted as AMPs with an extremely high probability score (<0.99). Some physicochemical properties of the peptides, including size, charge, hydrophobic ratio and moment, Bowman index, and instability and aliphatic indices, were considered to further reduce the number of potential candidates before undergoing molecular dynamics (MD) simulations. All molecules with less than 4 AA or more than 30 AA were disregarded, considering the average size of most potent already reported AMPs. Furthermore, since most AMPs typically interact with negatively charged membranes of microorganisms, only cationic peptides were taken into account [35].

Additionally, to enable a transmembrane and phospholipid-peptide interaction, it has been recommended that the hydrophobic ratio and hydrophobic moment must have values of at least 0.4 and less than 0.3, respectively [69,70]. The Eisenberg Scale was used to calculate both indexes. Moreover, given the disruption-membrane capability of most AMPs, the isoelectric point of AMPs has been reported near 10, which is similar to that of soap or detergents [70,71,72]. Finally, the Boman Index estimates the potential of peptides to bind to other proteins. A low index value suggests that the peptide is likely to exhibit a high antimicrobial activity without promoting significant side effects. A higher index value indicates that the peptide is likely to have multifunctional roles within the cell, leading to higher chances of side effects [73,74]. Therefore, intermediate Boman indexes are desirable to balance toxicity and broad-spectrum activity.

**Figure 5:**
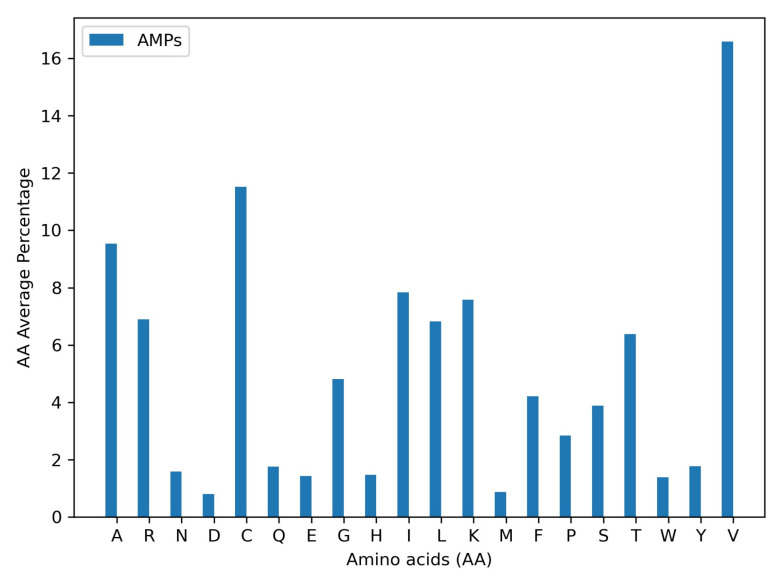
Average percentage of each amino acid within all sequences of predicted AMPs.

Likewise, stability, as determined through the instability and aliphatic indices, is an essential characteristic of therapeutic peptides. In this regard, peptides are considered unstable when the instability index is above 40, which is related to low bioavailability and short half-life [75]. On the contrary, for the aliphatic index, which is related to heat stability, the higher its value, the higher the heat stability the peptide will exhibit [76]. After filtering the peptide candidates with such criteria, we found 252 that fulfilled all of them. Figure 5 shows their amino acid distribution.

The predicted AMPs are rich in lysine (K), cysteine (C), and arginine (R), which correlates well with previous reports available in the literature [55]. Somewhat surprisingly, valine was the most abundant AA in the sequences. Even though it has not been reported as the most prevalent AA in the database statistics, recent reports indicate its capability to enhance antimicrobial activity by increasing hydrophobicity. This strongly suggests that it is very likely that the DL model is capable of learning hidden patterns related to the underlying chemistry of peptides that pass undetected to humans. Even though 252 candidates are feasible for screening at the industrial scale, a further reduction was needed for the scope of this work.

#### 3.2.1. Monofunctional Peptides: AM Activity

To reduce the number of candidates, we performed a study of AM motifs based on a dermaseptin (AL) AMP extracted from a skin micro-organ of *Phyllomedusa bicolor*, previously evaluated in our laboratory [77]. The analysis predicted four possible AM motifs for the peptide: (i) LWKD, (ii) ALWK, (iii) WKDL, and (iv) LKKV. The first motif has been widely studied within dermaseptin peptides, and the second one is merely an extension of it [78,79,80,81,82]. The third one has also been identified and studied previously [83]. However, the fourth motif has not been reported previously, so we focused on the new sequences containing it. Conversely, we chose two promising candidates, KLKKVTGKKMSKCMKCKIYVCS(KS22) and VFVVVTLLKKVKLLC(VC15), which also showed Boman indexes that might lead to different interactions with microorganisms’ membranes.

#### 3.2.2. Bifuctional Peptides: AM + CP Activity

**Figure 6:**
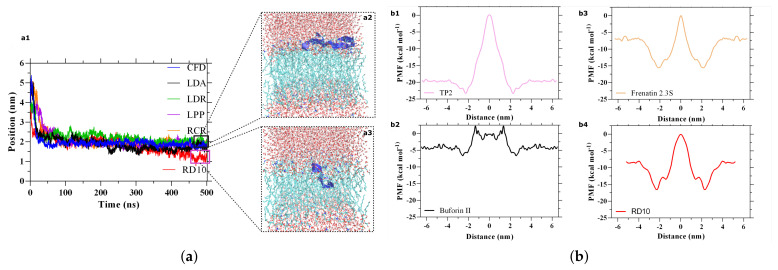
RD10 peptide interaction with a model lipid bilayer as estimated by Molecular Dynamic (MD) simulations. (**a**) Penetrating capability of multiple promising candidates. Only the RD10 peptide can penetrate the cellular membrane. (**b**) PMF profiles of RD10 and three already validated cell-translocating peptides in vitro. (**a**) Flat bottom. (**a1**) Peptides position traces within the simulation box for 500 ns. Cellular membrane positioned at 2.4 nm. Only the RD10 sequence is likely to penetrate the cellular membrane; however, the other candidates seem to interact with the headgroups of the phospholipid bilayer strongly and remain therein. (**a2**) Final position of the CFD peptide. (**a3**) In its final position, the RD10 peptide has completely penetrated the bilayer and is located deeper within the hydrophobic core. (**b**) Umbrella Sampling. Free energy profile (PMF) for peptides translocating a simplified eukaryotic membrane. Free energy profile (PMF) for peptides translocating a simplified eukaryotic membrane. (**b1**) TP2, a cell-penetrating peptide added for comparison. (**b2**,**b3**) The antimicrobial peptide Frenatin 2.3 and the antimicrobial, cell-penetrating, and DNA binding peptide Buforin II. Also added for comparison. (**b4**) RD10, an antimicrobial and cell-penetrating candidate.

We selected five random peptides within the 252 candidates with a BI of around 2.0 and a GRAVY index close to 0.2 for analysis via MD simulations to find possible cell-penetrating peptides. Their traces along 500 ns of simulation ((a1) in Figure 6a) showed significant membrane interactions but incapacity for translocation. Peptides move from the bulk (at around 5.0 nm) to about 2.4 nm along the *z*-axis of the simulation box where the membrane was located and remained semi-static throughout the simulation. This behavior has been reported previously for several AMPs: they first accumulate near the membrane before acting through various mechanisms, including carpeting, induction of non-lamellar lipid phases, or formation of discrete pores [84]. (a2) in Figure 6a shows the final position of the CFD peptide where it is strongly interacting with the head groups of the lipid bilayer, most likely through electrostatic interactions, but fails to penetrate it. Its parallel orientation on the membrane surface can be related to the carpet mechanism of membrane interaction exhibited by some cationic peptides [85].

Even though highly charged cationic peptides have been reported to penetrate cellular membranes, the energy requirement is significantly high depending on the underlying mechanism. Therefore, to enhance the probability of membrane translocation rather than disruption, we selected a peptide with a lower net charge [86,87]. In contrast to other peptide candidates, RTLFVCRVGD (RD10) (red in Figure 6a1) was able to penetrate the cellular membrane and locate deeper within it (Figure 6a3). We then conducted an Umbrella Sampling simulation to calculate the energy requirement needed for RD10 to translocate the membrane. Figure 6b shows the free energy profile (PMF) of the RD10 (b4) and three already validated and reported peptides, analyzed with the same MD simulation. Compared to Frenatin 2.3S (a cell-penetrating antimicrobial peptide extracted from skin micro-organs of the Orinoco Lime Treefrog), RD10 has a similar energy profile (b3) with an additional DNA binding capability. This result strongly suggests that RD10 should be able to translocate the membrane effectively [88]. Furthermore, given its lower ΔPMF compared with TP2(b1), a non-disruptive membrane-translocating sequence, RD10 should be able to enter the cell without causing integral damage to the membrane [89]. Finally, compared to Buforin II(B), a cell-penetrating antimicrobial peptide with DNA binding affinity, RD10 has a significantly higher energy requirement to penetrate the cell. This implies that RD10’s translocation efficiency is likely to be below Buforin II and that if trafficked intracellularly by endocytosis, it should remain trapped within endosomes to a larger extent [90].

**Figure 7:**
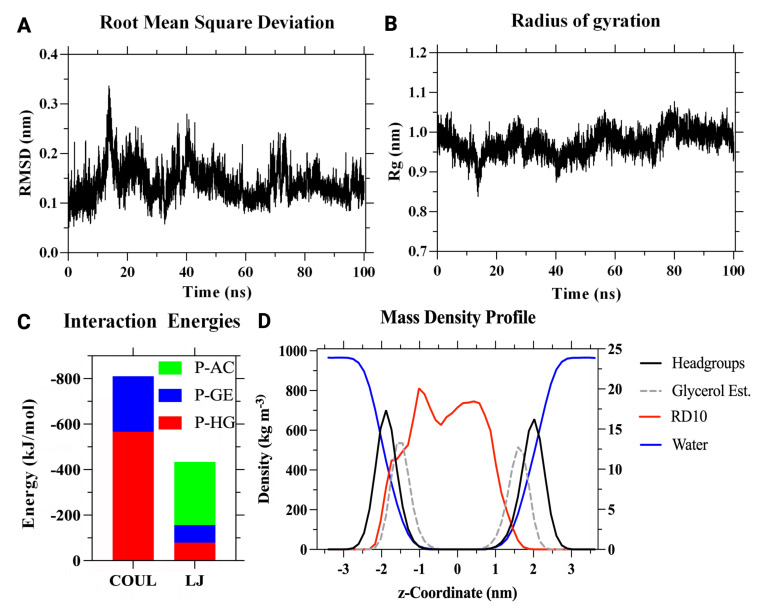
Stability of the RD10 peptide inside the cellular membrane. A minor variation on (**A**) RMSD and (**B**) Rg indicates that the peptide maintains its 3D conformation along the translocation process. (**C**) Coulombic (COUL) energies dominate the interactions over Lennard-Jones (LJ) energies. Headgroups (P-HG) and acyl chains (P-AC) play a significant role in peptide-membrane interaction. (**D**) Density distribution of the peptide along the *z*-axis of the membrane. The right *y*-axis presents the density scale for peptides, while the left *y*-axis represents the membrane components and bulk water. It indicates that the peptide remained mainly within the acyl chains, with a minor fraction interacting with the headgroups.

Given the slight variations in RMSD and RG (Figure 7A,B), RD10 is likely to preserve a stable 3D conformation along the translocation process, specifically its folded structure and its globularity [91,92,93,94]. The membrane was deconstructed into headgroups, glycerol ester, and acyl chains. Their distribution, the peptides, and water were determined in the z-direction, perpendicular to the surface of the bilayer, as shown in the density profiles of Figure 7D. No alteration or asymmetry was observed in the profiles. The peptide remained mainly within the acyl chains; however, also a portion interacted with the headgroups. Those interactions are corroborated by the interaction energies, which indicate an electrostatic binding mechanism (coulomb energy) driven mainly by the peptides’ interaction with the partial charges of the head groups (P-HG). Alternatively, the Lennard Jones (LJ) potential energies suggest that the most significant interaction is with the acyl chains (P-AC) (Figure 7C). Overall, the MD results strongly indicate that RD10 is a promising candidate for membrane translocation.

To choose another promising peptide candidate, we performed a motifs analysis on RD10 and discovered the FVCR motif, which has not been reported previously. We selected one of the two possible candidates that share such a motif. Selected peptides for in vitro validations and their corresponding physicochemical properties are shown in Table 7. All peptides but FTFYLPLFVCRRNPRPRRVSCRE (FE23) fulfilled the metadata requirements; however, it was chosen due to its unique motif and compliance with most of the desired physicochemical properties. Furthermore, it was also taken into consideration that stability and bioavailability can be enhanced further through subsequent biochemical modifications after experimental validation confirms its potential. Finally, to asses AMPs-Net’s performance towards non-AMP peptides, we evaluated AHB-1 (MFVFLVLLPLVS), a potent membrane-transloacting peptide that was recently discovered by us from a comprehensive analysis of the spike protein of SARS-CoV-2. AHB-1 is predicted as non-AMP with a probability of 99.7%. Its physicochemical properties are shown in Supplementary Table S1.

### 3.3. AM Validation

The minimum inhibitory concentration (MIC) assay was performed for KS22, RD10, and FE23 (Table 8). VC15 was not thoroughly evaluated due to solubility issues both in organic and aqueous media. The validation was performed in media containing both Na${}_{2}$PHO${}_{4}$ and NaCl due to the insolubility of KS22 and FE23 in Na${}_{2}$PHO${}_{4}$. Both FE23 and KS22 exhibited a bactericidal activity, while RD10 exhibited a bacteriostatic activity in Na${}_{2}$PHO${}_{4}$. FE23 has the most significant activity against both *E. coli* and *S. aureaus*, with MIC values of 7.8 μM and 15.63 μM, respectively. These results are comparable with the most effective AMPs described in the literature, in which MIC values range from 0.125 to 16 μM [95]. However, it is important to note that the insolubility might be reducing the performance of both FE23 and KS22, leading to higher MIC values compared to a scenario where the peptides would be completely soluble [96,97]. In this regard, even though KS22 has a relatively high MIC value, it inhibits 50% of bacterial growth starting at 7.8 μM for both evaluated strains. Likewise, RD10 inhibits 50% of bacterial growth starting at 0.48 μM for *E. coli* and 3.9 μM for *S. aureus*. However, this peptide showed no solubility issues that might potentially decrease its activity at higher concentrations. Therefore, RD10 can be considered a bacteriostatic peptide as it reduces bacterial cellular activity but is incapable of causing bacterial death [98]. A similar situation was observed for the NaCl assay, where no MIC values were recoverable. However, 50% of inhibition was observed at the same low concentrations. This behavior correlates well with the results of MD simulations since it has been reported that highly bactericidal AMPs usually exhibit high membrane activity, but not necessarily translocation capacity [99]. It is important to highlight that RD10 is likely to exhibit less selectivity towards microbes than mammalian cells due to its low charge, thereby limiting its possible use in clinical applications [100]. Nevertheless, this can only be confirmed by standardized cytotoxicity, hemolytic, and platelet aggregation assays, which are mandatory before moving to pre-clinical validation scenarios.

On the contrary, both FE23 and KS22 significantly reduce their antibacterial activity when evaluated in NaCl. This reduction is likely related to the presence of ions that might block the active moieties involved in the interaction with the bacterial membranes. In this regard, salts have been reported to reduce the peptide-membrane initial binding event’s kinetics, especially in highly cationic peptides, such as FE23 and KS22. An increased NaCl concentration might shield the charges responsible for interaction, resulting in a delayed binding process [101]. This phenomenon occurs due to sodium ions binding on deep sites within the lipids’ head group region, leading to the complexation of lipids and a decrease in the average area available for the peptide intermingling and final disposition within the bilayer. Since highly cationic AMPs are likely to have a similar interaction mechanism, salt cations compete for the same interaction sites, reducing the possibility of triggering any possible damage to bacterial membranes. In other words, the closer packing of the lipids in the presence of salt molecules leads to less potential destabilization of the lipid bilayer by the membrane-active peptides [101,102].

Even though we failed to find MIC values for FE23 and KS22 when evaluated in NaCl, growth-inhibitory activity was observed for both peptides (Table 9). KS22 inhibits 50% of growth at 125 μM for *E. coli* and 31.25 μM for *S. aureus*. Likewise, FE inhibits 50% of the growth of *S. aureus* at 125 μM; however, we failed to find the corresponding value for *E. coli*. The higher activity of KS22 compared with FE23 (which showed higher activity in Na${}_{2}$PHO${}_{4}$) is likely related to a stronger competition of sodium cations with less positively-charged peptides. Given KS22’s higher charge, it has more affinity towards the membrane’s lipids’ head group region, with a lower binding time for the peptide-membrane complex. Therefore, when exposed to NaCl under the same conditions, the reduction of the antibacterial effect is more marked in peptides with lower charges, specifically, FE23.

The differences in activity observed for the peptides towards the two different types of bacteria evaluated (i.e., gram-negative vs. gram-positive) is most likely related to the differences in bacterial surface charge distribution. This results in more or less inhibition by interactions with the salt cations present in the medium [103].

Overall, the proposed DL model correctly predicted the AMP activity of the three peptides; however, the obtained probability scores failed to show a correlation with the relative potency of the peptides. FE23, KS22, and RD10 obtained scores of 0.9937, 0.9928, and 0.9916, which indicated a similar potency that was not observed experimentally. Nonetheless, it is important to keep in mind that the binary model considers not only antibacterial activity but also antiviral, antifungal, and antiparasitic activities. Therefore, RD may have a higher potency towards other microorganisms that were not in the scope of the present contribution. This hypothesis appears further corroborated in silico with the aid of a multilabel model, where the highest predicted activity for RD was antiviral with a probability score of 0.7103. Furthermore, AMPs-Net specific model correctly predicted the bacteriostatic activity of both FE23 and KS22 with probability scores of 0.7066 and 0.7584, respectively. Overall, the antibacterial assays validated our models’ potential to screen peptides in silico and reduce the most promising candidates to a few that can be validated experimentally, thereby saving time and other valuable resources.

As an additional validation of the predicting capacity of AMPs-Net, we performed the same MIC assay with AHB-1. Even though the peptide showed no solubility issues, we failed to find the MIC value for any of the evaluated bacterial strains. Furthermore, no growth inhibition was observed even at the highest concentration evaluated of the peptide, i.e., 250 μM (Supplementary Table S2). The non-AM activity goes in accordance with the prediction of AMPs-Net. More details are available in the supplementary material.

### 3.4. CP Validation

**Figure 8:**
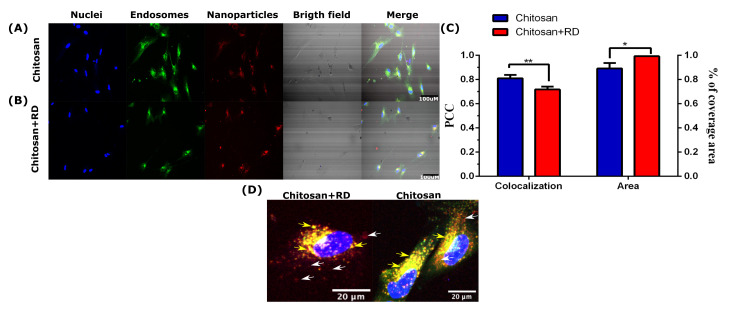
Cell-Penetration assay. (**A**) Internalization of bare CNPs into NHA cells (**B**) and CNPs-RD10 nanobioconjugates (20X magnification, 3 h of exposure). The scale bar corresponds to 100 μM. (**C**) Quantification of colocalization between CNPs and CNPs-RD10 nanobioconjugates by the Pearson correlation coefficient (PCC) and the fraction of cytosol area covered by CNPs and CNPs-RD10 nanobioconjugates. There is a statistically significant difference between both treatments. *p* ≤ 0.05 (*), *p* ≤ 0.01 (**). (**D**) Visual inspection of colocalization studies via confocal imaging. Yellow arrows point to colocalization regions between the green and the red channels, showing CNPs or CNPs-RD10 nanobioconjugates trapped in endosomes. The white arrows indicate non-colocalized regions where CNPs or CNPs-RD10 nanobioconjugates likely escaped endosomes or reached the intracellular space by different internalization mechanisms.

The Pearson correlation coefficient (PCC) was used here to quantify colocalization with endosomal compartments and the percentage of area covered as a measure of the CNPs-peptide nanobioconjugates that internalized cells successfully. Figure 8A,B,D compare the internalization of bare chitosan nanoparticles (CNPs) and RD10 immobilized on CNPs (CNPs-RD10 nanobioconjugates). The red fluorescent signal confirms the internalization of CNPs and CNPs-RD10 nanobioconjugates; however, the merged channel shows high colocalization levels with Lysotracker Green in both cases. CNPs-RD10 nanobioconjugates show more non-colocalized regions than bare CNps, which confirms the role of RD10 as a potent membrane-translocating peptide (Figure 8D). The colocalization of bare CNPs with endosomes appears to have distributed all along the cytosol, while in the case of the nanobioconjugates, they seemed to cluster in specific regions. These qualitative observations were confirmed quantitatively with both the PCC and the percentage of area covered by the nanobioconjugates (Figure 8C), as evidenced by a statistically significant difference in both metrics between bare CNPs and CNPs-RD10 nanobioconjugates. CNPs-RD10 nanobioconjugates reached a percentage of the covered area of 99.31 ± 0.5, while that of bare CNPs was 89.16 ± 4.4. Additionally, the PCC for the CNPs-RD10 nanobioconjugates approached 0.72 ± 0.02, while it was 0.81 ± 0.03 for the bare CNPs. Taken together, both results support the notion that once internalized, the CNPs-RD10 nanobioconjugates escaped endosomes to a larger extent, reaching a higher cytosol coverage. Also, given the relatively low positive charge of RD10, it is likely that the most plausible mechanism for endosomal escape is direct translocation [104]. Further analyses need to be carried out to elucidate the mechanistic details of internalization and intracellular trafficking. All in all, our findings confirm that the discovery approach introduced here is well-suited for finding new and more potent membrane-active peptides.

### 3.5. Clinical Applications

Along with the development of antibiotics, bactericidal agents have been presumed superior to bacteriostatic agents. However, in clinical practice, bacteriostatic pharmaceuticals have been used to treat effectively multiple gram-positive bacterial infections and are even preferred in some cases where the sudden death of the infecting bacteria might lead to the release of exotoxins that can be dangerous for the patient [105]. Furthermore, there is no solid evidence in the literature that corroborates that bactericidal antibiotics are more effective clinically than bacteriostatic agents [106]. Therefore, the preliminary results shown here for RD10 make it a promising candidate for further biocompatibility and antimicrobial testing prior to pre-clinical and clinical trials. Specifically, it would be of particular interest to evaluate its antiviral activity, which our model predicted as the most important. Moreover, RD10 holds much promise in the pharmaceutical arena mainly due to its bifunctional activity, which could be helpful in multiple clinical scenarios, including cancer therapy, obesity, and neurodegenerative diseases treatment. [12,14,15,16,17,18,19,20].

In contrast, FE23 and KS22 have a much lower potential for clinical usage because of their low solubility in buffers with compositions similar to that of plasma and their high sensitivity to salts also present in plasma and blood. Even though in some cases the loss of activity in media containing salts could be overcome by larger exposure times, this solubility issue demonstrates that our discovery pipeline requires further optimization to find clinically relevant candidates. For instance, our models could only select as active candidates those with relatively low MIC values in NaCl (0.9% *w*/*v*), which might be a challenging task considering that such information might be scarce.

Additionally, a more rational design would be needed to overcome the solubility issues encountered. The IA models within our pipeline would focus on finding bioactive motifs instead of complete sequences. Considering that the most stable peptides in buffers containing similar salts concentrations of human plasma and blood might be obtained from marine life [97], one could use the non-motif regions of the peptides from marine life as templates to merge with the motifs found by IA. It is important to consider that the relative position of these two regions within the peptide sequence is vital to maintaining or losing the bioactivity of the original peptides.

Finally, the solubility of peptides might be enhanced by their immobilization on nanomaterials modified with polyethylene glycol, a polymer that has been widely used to increase the solubility and stability of numerous biomolecules [107,108]. Thus, the modified nanobioconjugates could exhibit improved solubility, stability against degradation, increased circulation times, and prolonged biological activity [109]. Furthermore, it has been reported that some peptides significantly increased their activity against gram-positive and gram-negative bacteria upon immobilization on nanomaterials compared with free AMPs [110,111]. Therefore, the activities of FE23 and KS22 could be enhanced following either of the proposed approaches and therefore re-evaluate whether they could eventually undergo further testing to reach pre-clinical and clinical stages.

## 4. Conclusions

To the best of our knowledge, we proposed a new database that has a more significant number of antimicrobial peptides experimentally validated than other previously recollected datasets. Our AMPs-Net outperforms all deep learning methods proposed thus far and pioneers the implementation of graph representations to precisely describe the relationship between atoms, which is fundamental for predicting peptides’ bioactivities more accurately. Furthermore, besides the superior capacity of AMPs-Net for specific antibacterial and antiviral activity prediction, it is the first deep learning model to study the four main antimicrobial activities simultaneously.

Additionally, bioprospection of a peptide library derived from *E. coli*’s fragmented genome through graph convolutional networks was performed to identify new antimicrobial peptide candidates. Three out of the four selected peptides were evaluated in vitro, and all presented antimicrobial activity, therefore validating the virtual screening conducted with the aid of our artificial intelligence algorithms. Furthermore, through molecular dynamics simulations, we investigated in more detail the peptide-membrane interactions of novel molecules and hypothesized possible mechanisms of action that will be studied in future work. Our results show that the components of our proposed discovery pipeline work properly and can be further optimized to enhance the velocity at which AMPs discovery might take place as more experimental data become available.

RD10 was validated as a multifunctional bacteriostatic peptide and is a promising candidate to study further as an antiviral molecule. Additionally, its biocompatibility needs to be tested comprehensively via standardized tests in line with the ISO-10993 standard, which is mandatory prior to move to pre-clinical and clinical assays. Even though the developed neural networks correctly predicted the AM activity of the peptide sequences FE23 and KS22, some crucial physicochemical properties related to solubility and stability in physiological media (primarily due to the presence of salts) were disregarded in the model’s design. This resulted in a significant loss of activity; however, we proposed to address this issue in future work by engineering new sequences through a combination of active and already known stable motifs derived from proteins of marine organisms. This can be complemented by their immobilization on nanostructured materials as this approach has been reported to favor half- and shelf-life and thermal stability for numerous therapeutic proteins.

Notably, the significant reduction in the candidate molecules to test experimentally (only 252) is advantageous for biopharmaceutical companies whose high-throughput screening platforms can complete such tasks very rapidly and at a meager cost. All in all, our work provides an appealing route for AMPs discovery that relies on a relatively inexpensive computational framework that can be further optimized by running only selected experiments.

## Figures and Tables

**Table 1:** Databases recollected to perform the IA model’s training. ${}^{1}$ The database consists of 8305 sequences; however, some are proteins, which are not of interest for this work.

Database Name	Number of Peptides
BIOPEP-UWM Database [37]	3634
CPPsite 2.0 [38]	1155
CAMP${}_{R3}\;{}^{1}$ [39]	4519
TumorHoPe [40]	787
APD3 [41]	3072
SPdb [42]	2512
ParaPep [43]	194
CancerPPD [44]	556
BrainPreps [45]	92
Quorumpeps [46]	257
YADAMP [47]	2525
LAMP2 [48]	5454
Milkampdb [49]	260
DADP [50]	2557
AntiTbPdb [51]	271
PeptideDB [52]	1903
NeuroPrep [53]	3875
SATPdb [54]	9664
Other peptides	1475
Total	44,762

**Table 2:** Distribution of biological activities of peptides within the database.

Biological Activity	Number of Peptides
Antimicrobial	13,468
Neuropeptide	3615
Signal-Peptide	2351
Anuran-Defense	1783
Anticancer	1602
Cell-Penetrating	1155
ACE-Inhibitor	934
TumorHoming	704
Antioxidative	637
Peptidase-IV-Inhibitor	420
Toxic	256
QuorumSensing-Peptide	252
Opioid	136
BBB-Peptide	88
Immunomodulating	71
Peptidase-III-Inhibitor	66
Haemolytic	63
Antithrombotic	58
Antiamnestic	52
CaMKII-Inhibitor	50
Insecticidal	49
Alpha-glucosidase-Inhibitor	34
Renin-Inhibitor	19

**Table 3:** Atom and bond features description. Feature vectors were obtained with the aid of the RdKit and OGB libraries, which describe the state of an atom and a bond within a molecule.

Atom Features	
Atomic Number	1, 2, …, 119
Chirality	Unspecified, Tetrahedral clockwise, Tetrahedral anti-clockwise, Other
Degree	0, 1, …, 10
Formal Charge	−5, −4, …, 4, 5
Number of Hydrogens	0, 1, …, 8
Number of radical e${}^{-}$	0, 1, …, 4
Hybridization	Sp, Sp${}^{2}$, Sp${}^{3}$, Sp${}^{3}$d, Sp${}^{3}$d${}^{2}$
Aromaticity	0, 1
Ring membership	0, 1
**Bond Features**	
Type	Single, Double, Triple, Aromatic
Stereochemistry	None, Z, E, CIS, TRANS, Any
Conjugation	0, 1

**Table 4:** Comparison with SOTA. We compare AMPs-Net with multiple state-of-the-art deep learning methods and the best Random Forest algorithm. For comparison with DL methods, pre-trained models or prediction servers were used; no retraining was possible due to a lack of public or non-working codes. ${}^{1}$ Prediction servers failed to return the probability scores; therefore, we failed to calculate AP. ${}^{2}$ Best Random Forest algorithm to date as it was retrained with our data.

Method	AP	ACC
AMPScanner [32]	82.1	65.58
AI4AMPs [64]	76.74	67.64
CAMP${}_{R3}$ ${}^{1}$ [65]	-	67.82
AMPDiscover ${}^{1}$ [66,67]	-	71.63
AMPlify [30]	86.96	75.07
AMPs-Net (Ours)	95.76	89.81
Non-deep learning SOTA		
AMPEPpy (RF) ${}^{2}$ [68]	97.37	90.33

**Table 5:** Binary AMPs-Net performance. Optimization of the GCN Module. In bold best configuration attained.

Parameter	Test AP
GCN layers	
10 Layers	94.48
15 Layers	94.32
20 Layers	95.04
25 Layers	94.86
30 Layers	94.6
Features Hidden Size	
HS 32	91.52
HS 64	94.12
HS 128	94.67
HS 256	95.04
Aggregation Function MLPs	
2 MLP	94.72
3 MLP	95.04
4 MLP	95.09
Metadata concatenation	
**8 Features**	**95.76**

**Table 6:** Multilabel model performance.

Antimicrobial Activity	AP
Antibacterial	90.57
Antiviral	84.54
Antifungal	50.93
Antiparasitic	24.73
**Overall evaluation**	**NAP**
Multiclass	71.36

**Table 7:** Physicochemical properties of selected peptides. Values for each selection criteria on the four peptides selected for in vitro experimentation. ${}^{1}$ Monofunctional peptides: AMPs. ${}^{2}$ Bifunctional peptide: AMP + CPP.

Sequence	Size	Net Charge	Boman Index	Hydrophobic Ratio	Hydrophobic Moment	Aliphatic Index	Instability Index	Isoelectric Point
VFVVVTLLKKVKLLC ${}^{1}$ (VC15)	15	2.834	−1.661	0.733	0.257	200.666	−15.226	10.425
KLKKVTGKKMSKCMKCKIYVCS ${}^{1}$ (KS22)	22	7.521	1.205	0.409	0.244	61.818	32.141	10.527
RTLFVCRVGD ${}^{2}$ (RD10)	10	0.836	2.293	0.5	0.195	97.0	0.509	8.759
FTFYLPLFVCRRNPRPRRVSCRE ${}^{1}$ (FE23)	23	4.68	3.463	0.391	0.240	59.103	107.39	11.428

**Table 8:** Antimicrobial assay. MIC values for the four peptides in Na${}_{2}$PHO${}_{4}$ buffer and NaCl for Gram-negative *E. coli* and Gram-positive *S. aureus*.

Sequence	Na${}_{2}$PHO${}_{4}$		NaCl	
	MIC (μM)		MIC (μM)	
	*E. coli*	*S. aureus*	*E. coli*	*S. aureus*
VFVVVTLLKKVKLLC (VC15)	>160	>160	-	-
KLKKVTGKKMSKCMKCKIYVCS (KS22)	250	250	>250	>250
RTLFVCRVGD (RD10)	>250	>250	>250	>250
FTFYLPLFVCRRNPRPRRVSCRE (FE23)	7.8	15.62	>250	>250

**Table 9:** Inhibition in Bacterial Growth. Peptides’ concentration (μM) that have inhibitory effects on bacterial growth.

Sequence	Concentration (μM)	
	*E. coli*	*S. aureus*
VFVVVTLLKKVKLLC (VC15)	80	80
KLKKVTGKKMSKCMKCKIYVCS (KS22)	7.8	7.8
RTLFVCRVGD (RD10)	0.48	0.48
FTFYLPLFVCRRNPRPRRVSCRE (FE23)	3.9	3.9

## Data Availability

The database recollected for the development of AMPs-Net can be share under request to the first author. Code and pre-trained models will be made available at https://github.com/BCV-Uniandes/AMPs-Net (accessed on 6 June 2022).

## Supplementary Materials

The following supporting information can be downloaded at: https://www.mdpi.com/article/10.3390/membranes12070708/s1, Table S1: Physicochemical properties of AHB-1; Table S2: Antimicrobial assay. MIC values for AHB-1 peptide.
