# Supplementary material for: Rational Discovery of Antimicrobial Peptides by Means of Artificial Intelligence
Source: Membranes (Basel). 2022 Jul 14;12(7):708. doi: 10.3390/membranes12070708 (PMC9320227; doi:10.3390/membranes12070708)
Supplement: Supplementary file 1 [file membranes-12-00708-s001.zip › membranes-1793185-supplementary.pdf]

Supplementary Material for Rational discovery of antimicrobial peptides by means of artificial intelligence

Table S1. Physicochemical properties of AHB-1. Values for each selection criteria utilized to select the most promising candidates.

Sequence	Size	Net Charge	Boman Index	Hydrophobic Ratio	Hydrophobic Moment	Aliphatic Index	Instability Index	Isoelectric Point
MFVFLVLLPLVS	12	-0.01	-3.059	0.833	0.109	202.5	25.216	6.0

Table S2. Antimicrobial assay. MIC values for AHB-1 peptide in Na_2PHO_4 buffer for Gram-negative *E. coli* and Gram-positive *S. aureus*.

Sequence	MIC(μM)	
	E. coli	*S. aureus*
MFVFLVLLPLVS	> 250	> 250

Following Table S1 we can see that AHB-1 fulfills multiple of the selection criteria for AM peptides; however, AMPs-Net was able to predict it as a non-AMP accurately. A MIC assay corroborated its bioactivity. AHB-1 has no bactericidal nor bacteriostatic activity toward *E. coli* or *S. aureus*. No growth inhibition was observed even at the highest evaluated peptide concentration.
